# Plasma and fecal hormone profiles in an Endangered, oviparous colubrid, the Louisiana pinesnake

**DOI:** 10.1371/journal.pone.0327193

**Published:** 2025-07-01

**Authors:** M.M. Richter, B.M. Roberts, M.R. Sandfoss, S.B. Reichling

**Affiliations:** 1 Department of Conservation and Research, Memphis Zoo, Memphis, Tennessee, United States of America; Tshwane University of Technology, SOUTH AFRICA

## Abstract

Reptiles are sorely under-represented in endocrinology research. The majority of studies are conducted on lizards and turtles, rarely are oviparous snakes investigated. We utilized our breeding population of captive Louisiana pinesnakes (*Pituophis ruthveni*, LPS) to describe annual hormone cycles in an egg-laying colubrid. We collected fecal and blood samples from adult male and female snakes (20M.23F) throughout the year. After validating extraction methods and assays, we measured four hormones (corticosterone, estradiol, progesterone, and testosterone) in samples collected (200 + fecal and 600 + blood samples) over a two-year period. While blood samples were collected on a schedule, fecal samples were collected opportunistically. We found differences in patterns exhibited by males and females and between sample types. In females, neither fecal nor plasma samples showed significant differences between any of the collection periods, excepting increased levels found in female plasma progesterone PreLay compared to PostLay (n = 4 animals, p = 0.0323) demonstrating the importance of circulating progesterone in oviparous snake reproduction. In males, time played a significant role in fecal corticosterone levels (p = 0.0164). Male plasma showed a number of significant changes throughout the year: a significant increase from Post-Brumation to Breeding levels of corticosterone (p = 0.0058), Breeding estradiol and testosterone levels significantly higher (E2: p < 0.036, T: p < 0.005) than all other time bins except Post-Brumation (E2: p = 0.062, T: p = 0.1). Comparing differences and similarities between two different sample types, there is a clear advantage to collecting blood on a set schedule; we were able to analyze samples based on significant life history events and maintain larger sample sizes which may have contributed to the lack of differences measured in fecal hormone levels. This study helps to better understand the seasonal hormonal patterns in egg-laying snakes, and to aid in the recovery of this endangered species.

## Introduction

Steroid hormones are responsible for many important physiological events, such as growth and reproduction, in a vertebrate’s life and are commonly measured in seasonal and reproductive studies. Published studies on vertebrate hormone cycles largely skew to mammalian and avian species, with far fewer studies conducted on reptilian species. The multiple reproductive modes observed in reptiles, from viviparity to oviparity [1], requires in-depth study across Reptilia to identify both commonalities and differences if we are to understand selective mechanisms and evolutionary origins [2–4]. Despite oviparity being the most common reproductive method for reptiles (approx. 85% of reptiles are oviparous [5]; 80% of squamates are oviparous [6]), the majority of studies on snake hormones describe species that are viviparous, leaving a large gap in our knowledge.

The Louisiana pinesnake (*Pituophis ruthveni*, LPS) is a large, oviparous Colubrid endemic to the Southeastern United States. It is listed as threatened under the Endangered Species Act (2018; 83 FR 14958) and a species recovery plan has been established that includes captive breeding and release of hatchlings onto the landscape. Due to this captive breeding program, the Memphis Zoo houses 100 + adult snakes that undergo seasonal changes in temperature and photoperiod designed to mimic their natural environment and encourage successful breeding. As such, this large captive population provides the opportunity to answer questions regarding the seasonal fluctuations of adrenal and gonadal hormones in an oviparous snake species.

Glucocorticoids, such as corticosterone (CC) are produced by the adrenal glands and are implicated in mediating life-history trade-offs [7]. Elevated glucocorticoid levels are associated with physiological stress and play a role in both mobilizing fat stores as well as in muscle catabolism [8] and have been shown to correlate with reproduction in some reptiles [9,10]. Gonadal steroids such as estradiol (E2), progesterone (P4), and testosterone (T) play roles in the regulation of reproduction and are well-studied in endothermic vertebrates but to date, studies in reptiles, particularly oviparous snakes, are lacking. Estrogens (e.g., E2) are linked to sexual behavior and vitellogenesis in females and androgens (e.g. T) are associated with male sexual behavior and spermatogenesis [11]. In viviparous reptiles, progestins (e.g. P4) are responsible for maintaining pregnancy [12] while in turtles P4 acts to suppress E2-induced vitellogenesis (*Chrysmemys picta* [13]) and in other oviparous reptiles (e.g. crocodile and tuatara), P4 secretion from the corpus luteum only occurs during egg-shelling [14,15].

Recently, the role of hormones in influencing reproductive activity and timing in snakes has been used for management and conservation purposes. Researchers on Guam have attempted to manipulate hormone levels in invasive Brown treensnakes (*Boiga irregularis*) to suppress reproduction [16]. While, Parker and Mason feminized male garter snakes using E2 implants (*Thamnophis sirtalis parietalis* [17]) and artificially induced male-male reproductive behavior. In species recovery programs, hormone manipulation to stimulate reproductive activity has been successfully used in mammals and amphibians [18,19] but no similar examples exist for reptiles. This is probably the result of a lack of knowledge on hormone levels for the majority of reptiles [4]. This study aimed to help fill this gap in knowledge.

We collected fecal and plasma samples from both male and female adult LPS to outline annual hormone profiles for this species with a particular focus on reproductive cycling. We measured four steroid hormones commonly associated with seasonal changes: corticosterone (CC), estradiol (E2), progesterone (P4), and testosterone (T). We hypothesized that we would find seasonal differences in each hormone, between the sexes, but not necessarily between fecal and plasma samples collected during the same time periods. We predicted that these differences in hormone levels would correlate with specific reproductive physiological changes observed in our population. This is one of the only studies on hormone profiles in oviparous snakes, and the only study to-date to explore the advantages and disadvantages of collecting plasma compared to fecal samples in a snake species.

## Materials and methods

### Study population

The Endangered Louisiana pinesnake is a large bodied, oviparous colubrid endemic to Southwest Louisiana and parts of Texas, with a decreasing population according to the IUCN Red List [20]. The Memphis Zoo is involved in a multi-institute breeding and release program to recover this species within its historical range. As a member of this program, the Memphis Zoo has a large breeding colony of adult LPS that are kept on a naturalistic temperature and light regime across the calendar year. This study protocol was approved by the Institutional Care and Use Committee of the Memphis Zoo (Protocol Number: 2020−004).

In this study, we collected samples from 20 adult male and 23 adult female captive Louisiana pinesnakes. Animals are housed in a species-only building at the Memphis Zoo. Each animal was housed individually (outside of the breeding season (April to June) when 1 male was frequently housed with 1 female). Snakes experienced seasonal changes in light and temperature to mimic the natural environment including a simulated brumation period from 1 December to 1 March, with decreased ambient temperature from 27.7 ± 2°C to 10 ± 2°C and all artificial light removed. Animals were fed weekly from 15 March through 1 November; and had *ad libitum* access to water throughout the year.

### Fecal samples

From 2020 through 2021 fresh (<24h old) fecal samples were opportunistically collected from cages of individually housed animals. Some limitations found to systematic fecal collection include: samples found in the animal’s water bowl, samples that were dried out due to age and ambient conditions, as well as animals refusing food and thus not eliminating waste, particularly for males during breeding season and for gravid females. 200 + total samples were collected over this time period. Samples were labeled and stored frozen at −20°C until time of extraction. Samples were thawed at room temperature and any residual urates were separated out along with any undigested material, e.g. bone. Thawed fecal samples were placed in individual, labeled tins and dried in an oven (60°C) overnight (~16h). Once completely dry, samples were ground using a mortar and pestle, and any hair or bone fragments remaining were removed. A sub-sample was weighed out (0.25g) and combined with 50% methanol (5mL) at a ratio of 1:20. Tubes were rotated overnight (~16h), centrifuged (15 min at 2500 rpm), and the supernatant poured off and stored at −20°C until time of assay. Extracts were diluted 1:10 to 1:40, as needed, using the appropriate assay buffer for each hormone analyzed (details below).

### Plasma samples

Blood samples (0.5−1.0mL, n = 600 + samples) were attempted from the caudal tail vein (heparinized 22G, 1” needle) of non-anesthetized, hand-restrained snakes every two weeks during the active season in 2020 and 2021. Per our approved IACUC and with veterinary guidance, no analgesia was used as any potential suffering was minimized by using correct needle size, limiting draw attempts to 2 per time point, and expert procedure training. Plasma samples were not attempted from any animals actively breeding, from any animal that was opaque, nor from females that were heavily gravid and therefore could be injured during restraint. Time-to-collection for blood was recorded (mean = 4.9 ± 0.9 min) as was body temperature using a thermocouple temperature probe (Traceable Products™, Webster, TX, USA; #14-649-81) inserted ~3 cm into the cloaca. Blood samples were transferred to lithium heparin tubes immediately after collection to prevent clotting. Samples were centrifuged at 10,000 rpm for 10 min, the plasma was drawn off and stored at −80°C until time of extraction. Prior to assaying, samples were thawed at room temperature, and aliquoted (80−200 µL) into centrifuge tubes for protein precipitation using 600 µL of acetonitrile (Sigma-Aldrich, MO, USA), vortexed for 3minutes, and then centrifuged at 4-5k rpm for 3 min. The supernatant was drawn off and transferred to a new, clean centrifuge tube and dried completely using a heat block (38°C) and forced air. Dried samples were then stored frozen (−20°C) until reconstitution and assay. Samples were reconstituted at 1:2.5 or 1:5 using appropriate assay buffer (see below) with 5% methanol and a 20 min heated (30°C), sonicated bath prior to incubating overnight (~16h) at 4°C (technique modified from [21,22]). Reconstituted samples were diluted further with assay buffer as needed (up to 1:20) and assayed as described below.

### In-house assays

Concentrations of immunoreactive glucocorticoids, testosterone, estrogen, and progestagen metabolites in the fecal samples were analyzed using double antibody enzyme immunoassays (EIA) that crossreact to relevant metabolites [23,24]. The same assays were used to measure immunoreactive corticosterone, testosterone, and estradiol in the plasma samples; however, plasma progesterone was analyzed using a commercially available ELISA kit (Cayman Chemical Company, Ann Arbor, MI, USA; item #582601), following the manufacturer’s protocol and using the manufacturer’s assay buffer. Antibodies [mouse-anti-progesterone (CL425), rabbit-anti-corticosterone (CJM006), rabbit-anti-estradiol (R4972), rabbit-anti-testosterone (R156/7)] and corresponding steroid–3(O-carboxymethloxime) horseradish peroxidase (3CMO-HRP) conjugates were provided by C. Munro (University of California, Davis, CA).

Plates were coated with anti-rabbit IgG (10 µg/mL; Cat. No. A009, Arbor Assays, Ann Arbor, MI) or anti-mouse IgG (10 µg/ml; Cat. No. A008, Arbor Assays, Ann Arbor, MI) in coating buffer (Cat. No. X108, 20X, Arbor Assays, Ann Arbor, MI) by adding 150 µl to each well of a 96-well microtiter plate (Cat. No. 12-565- 135, Fisher Scientific, Pittsburgh, PA) followed by incubation at room temperature (RT) for 15–24 hours. All plates were blocked for 24 hours with blocking buffer (Cat. No. X109, 10X, Arbor Assays, Ann Arbor, MI) and then placed in a drying chamber until dry (relative humidity ≤20%), sealed in a bag, and stored at 4°C until time of assay. Plates used with corticosterone, testosterone, and estradiol were first coated with purified goat anti-rabbit IgG; plates used with progesterone (CL425) were coated with goat anti-mouse IgG. The dilutions of antibodies and horse-radish peroxidases were determined by checkboard titration. Corticosterone (CJM006), antiserum and horseradish peroxidase were used at dilutions of 1:175,000 and 1:275,000, respectively. Testosterone antibody (R156/7) was diluted 1:40,000 and 1:200,000 for Test-HRP; Estradiol (R4972) used 1:175,000–1:25,000 E2-HRP, Progesterone (CL425) used at 1:46,654 to 1:600,600 progesterone-HRP).

Day of assay, plates, samples, and all reagents were allowed to come to room temperature. Diluted samples and standards were added to the plate, followed by diluted antibody and horse-radish peroxidase. Loaded plates were incubated on a shaker (rpm 300) for 2 hours, prior to being washed. 100% TMB was used to develop the plate (10–45 min) and 1N HCl was used to stop the enzymatic reaction. Plates were read immediately on a MultiSkan FC plate reader (ThermoFisher. MA, USA) at 450nm. All assays were validated for each sample type using both a test of parallelism as well as standard recovery. The inter-assay variations were calculated based on the concentrations of control samples run on each plate, the intra-assay variation was calculated from the concentration CVs of the duplicates of each sample as follows: fecal corticosterone: 10.7% and 5.1%, plasma corticosterone: 5.5% and 5.3%; fecal estradiol: 14.04% and 5.8%, plasma estradiol: 12.4% and 10.6%; fecal progesterone: 8.0% and 4.0%, for plasma progesterone: 13.8% and 11.6%; for fecal testosterone: 13.1% and 7.8%, for plasma testosterone: 12.6% and 3.8%.

The cross-reactivities for the antibodies used are as follows: The corticosterone antibody (CJM006) cross-reacts with 100% with corticosterone, 14.25% with desoxycorticosterone and 0.9% with tetrahydrocorticosterone [25,26]. The polyclonal estradiol antibody (R4972) cross-reacts 100% with estradiol 17β, 3.3% with estrone, 1.0% with testosterone, 0.8% with progesterone, and less than 0.1% with the other steroids tested, including estrone sulfate, cortisol, corticosterone, and androstenedione [23,27]. The monoclonal progesterone antibody (CL425) cross-reacts with >50% with most 4-pregnene- and 5-a pregnan-metabolites [24]. The testosterone antibody (R156/7) cross-reacted (50% binding) with the following substances: 92.4% with DHT, 11.2% with 4-androsten 3_,17_-diol, 5.4% dehydroandrosterone, 3.4% androstanediol, 2.1% androstenedione, 0.5% androsterone, 0.4% epiandrosterone, 0.2% dehydroepiandrosterone, and less than 0.07% with hydrocortisone, cortisone, corticosterone, desoxycorticosterone, oestrone, oestradiol, progesterone, 17-alpha-hydroxy-progesterone, cholesterol and pregnenalone [28]. Per the manufacturer (Cayman Chemicals), the cross reactivity of their antibody is 100.0% for progesterone, 14.0% with pregnenolone, 7.2% with 17β-Estradiol, 6.7% with 5 β -pregnan-3α-ol-20-one, 3.6% with 17α-Hydroxyprogesterone and <1% with all other tested metabolites.

### Statistical analysis

All data presented are means ±SEM unless otherwise noted. Each sample type, hormone, and sex was analyzed separately using R Studio (version 4.3.2). Normality was determined using a Shapiro-Wilk test; data sets that were not normal were log transformed and re-tested for normality; normal data was analyzed using an ANOVA (significance: p ≤ 0.05) followed by a Wilcoxon rank sum pairwise comparison with Bonferroni post-hoc adjustment. Abnormal data was analyzed using a Friedman Test then a pairwise Wilcoxon rank sum test. Due to the different hormone time courses represented by the different sample types (plasma being reflective of the animal in that minute, fecal being reflective of the entire amount of time the sample was in the body, ~ 1wk in this species) as well as the frequency of collections, data was binned over slightly different times. For plasma samples, data was divided into time bins corresponding with observed life history events (PostBrumation: Julian day 0–80, Breeding: Julian day 86 to 125, PostBreeding: Julian day 190 to 220, Fall: Julian day 221 to 299, PreBrumation: Julian day 300 to 349); due to the limited availability of fecal samples, fecal hormone data was divided into 50-day bins that correspond to those same life-history stages for analysis (Julian Day 50–99 corresponding to PostBrumation;, Julian day 100–149 corresponding to Breeding, Julian day 150–199 and Julian day 200–249 corresponding to PostBreeding;, Julian day 250–299 corresponding to Fall, and Julian day 300–349 corresponding to PreBrumation). The slight differences in Julian dates help account for the delay in hormone deposition into feces compared to the minute to minute deposition in plasma.

## Results

### Male plasma hormones

**Fig 1:**
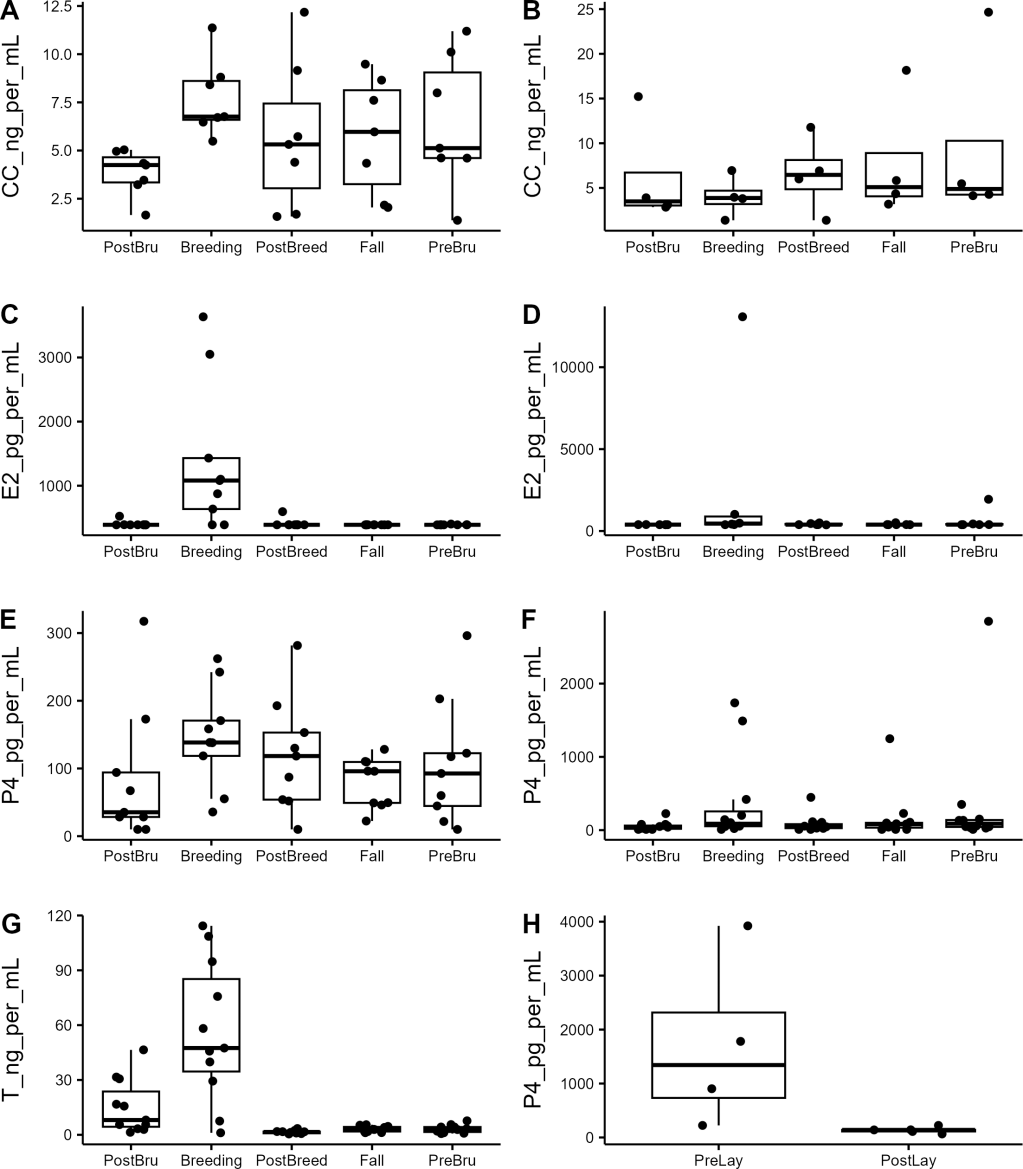
Plasma hormone levels from captive Louisiana pinesnakes across the active season. PostBru: Julian day 0−80, Breeding: Julian day 150−199, PostBreed: Julian day 190−299, Fall: Julian day 221−299. (A) Plasma corticosterone (CC, ng/mL, n = 7 individuals) for male snakes; no statistical patterns found. (B) Plasma estradiol (E2, pg/mL, n = 9 individuals) for male snakes; ‘Breeding’ significantly elevated compared to PostBreed, Fall, and PreBru (p < 0.05). (C) Plasma progesterone (P4, pg/mL, n = 9 individuals) for male snakes; no statistical patterns found. (D) Plasma testosterone (T, ng/mL, n = 11 individuals) for male snakes; levels were significantly higher during Breeding compared to Fall, PostBreed, and PreBru (p < 0.005) but not compared to PostBru; PostBreed T was lower than PostBru (p = 0.0064). (E) Plasma CC for 4 individual female snakes. (F) Plasma E2 for 6 individual female snakes. (G) Plasma P4 for 12 individual female snakes. (H) Plasma P4 before a clutch was laid (PreLay, within 14days of lay, n = 4 individuals) was significantly elevated (p = 0.03) compared to immediately PostLay (within 24h, n = 5 individuals).

Male plasma samples were analyzed for CC, E2, P4, and T levels. Corticosterone levels were log transformed to meet normality (n = 7 individuals, Shapiro-Wilk’s test W = 0.95856, p = 0.207, Fig 1A) and analyzed using an ANOVA which showed no significance in individual nor time bin (p > 0.05); a Wilcoxon Rank Sum test found a significant increase in plasma CC from PostBrumation to Breeding (from 3.8 ± 0.44ng/mL to 7.72 ± 0.75ng/mL, p = 0.0058). Estradiol levels were below the lower limit of detection for our assay for 7/9 individuals from the PostBrumation Bin, in 2/9 individuals from Breeding, for 8/9 individuals from the PostBreeding bin, in all 9 individuals assayed from Fall, and 8/9 samples from PreBrumation. Estradiol levels (n = 9 individuals, Fig 1b) were significantly elevated during Breeding (1398.61 ± 387.54pg/mL, p < 0.05) compared to all other time points except PostBrumation (405.14 ± 14.90pg/mL, p = 0.062). Plasma P4 levels (n = 9 individuals, Fig 1c) were found to have no significant differences across the year (Friedman Rank Sum chi-square = 0.93631, p = 0.2085). Testosterone (n = 11 individuals, Fig 1d) levels were significantly higher during Breeding (56.60 ± 11.51ng/mL) compared to Fall (3.11 ± 0.47ng/mL, p = 0.005), PostBreeding (1.52 ± 0.26 ng/mL, p = 0.005), or PreBrumation (3.14 ± 0.66ng/mL, p = 0.0039); PostBreeding T levels were significantly lower than PostBrumation (15.27 ± 4.48ng/mL, p = 0.0064) levels.

### Female plasma hormones

Female plasma samples were analyzed for CC, E2, and P4 levels. Corticosterone levels were log transformed to meet normality (n = 4, Shapiro-Wilk’s test, W = 0.94756, p = 0.3316, Fig 1e) and analyzed using an ANOVA, where no significance was found between groups (p = 0.794), individuals (p = 0.148) nor the interaction (p = 0.864). Estradiol levels did not show any significant changes throughout the year (n = 6, Friedman Rank Sum: chi-squared = 6.6484, p = 0.1557, Fig 1F). Progesterone levels trended towards an effect of time (n = 12, Friedman Rank Sum: chi-squared = 9.641, p = 0.04693, Fig 1G), but no time bins were found to be significantly different. There is a significant decrease (Welch Two Sample T-Test: t = 3.3676, p = 0.0323, Fig 1H) in plasma P4 levels within 24h post-lay (mean = 136.5 ± 28.9ng/mL, n = 5 individuals) compared to P4 levels prior to lay (within 14days, mean = 1707.7 ± 804.2pg/mL, n = 4 individuals). Due to limited sample volumes, we were unable to measure T in plasma samples collected from female snakes.

### Male fecal hormones

**Fig 2:**
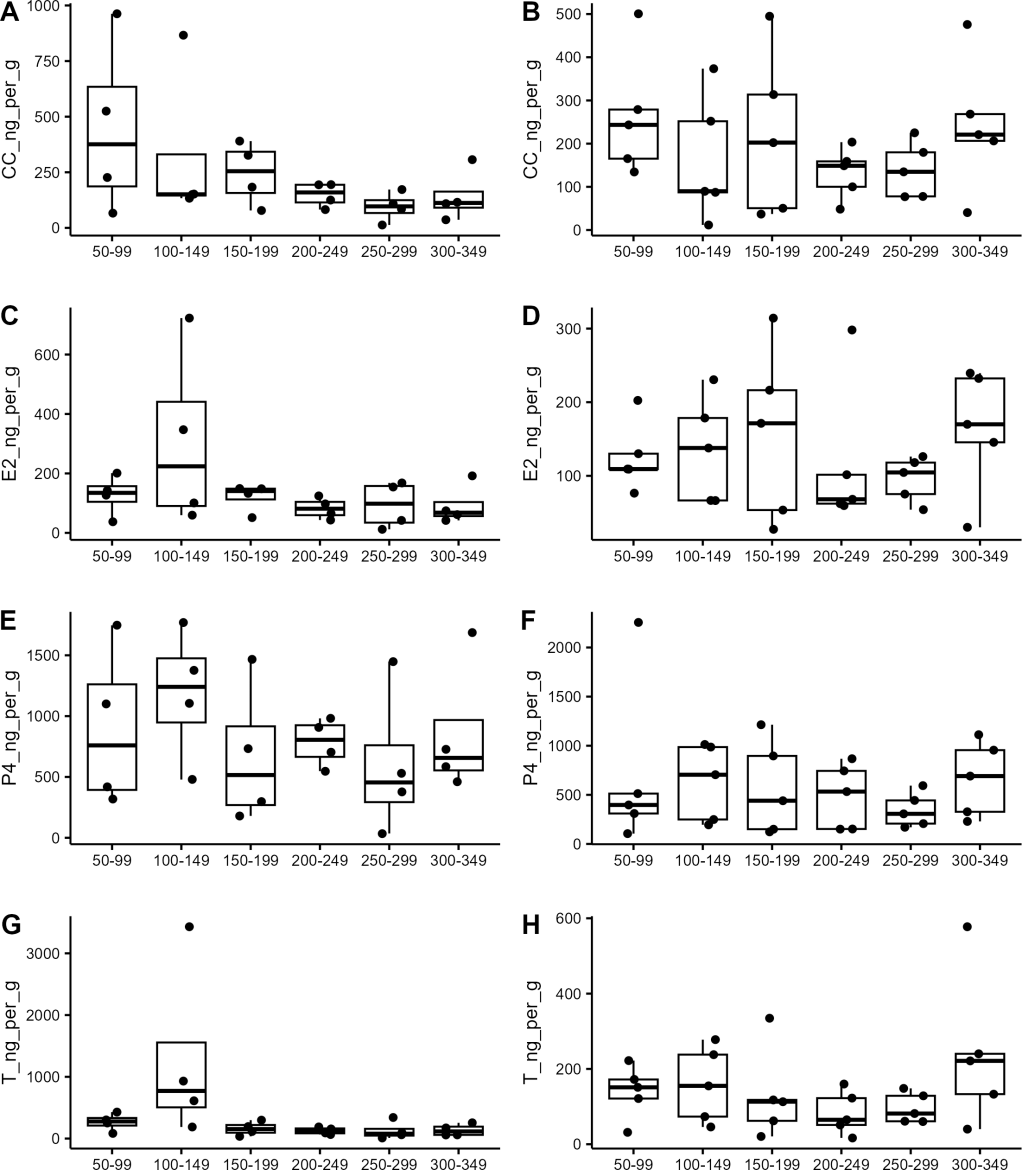
Fecal hormone levels from captive Louisiana pinesnakes across the active season. PostBru: Julian day 0-80, Breeding: Julian day 150-199, PostBreed: Julian day 190-299, Fall: Julian day 221-299. (A) Fecal corticosterone (CC, ng/g dried feces, n = 4 individuals) for male snakes. (B) Fecal estradiol (E2, ng/g dried feces, n = 4 individuals) for male snakes. (C) Fecal progesterone (P4, ng/g dried feces, n = 4 individuals) for male snakes. (D) Fecal testosterone (T, ng/g dried feces, n = 4 individuals) for male snakes. (E) Fecal CC levels for 5 individual female snakes. (F) Fecal E2 levels for 5 individual female snakes. (G) Fecal P4 levels for 5 individual female snakes. (H) Fecal P4 levels for 5 individual female snakes. No statistically significant patterns/differences were found for any hormones of either sex.

Male fecal samples were analyzed for CC, E2, P4, and T levels (Fig 2A, 2B, 2C, 2D respectively). Corticosterone levels showed a significant time effect (Fig 2A; n = 4, ANOVA: Day p = 0.0164), however no bin was significantly different from any other (Wilcoxon Rank Sum, p = 1.0). There were no significant effects of time on the other three hormones analyzed (n = 4, ANOVA E2: p = 0.615, P4: p = 0.441, T: p = 0.159).

### Female fecal hormones

Female fecal samples were analyzed for CC, E2, P4, and T levels (Fig 2E, 2F, 2G, and 2H respectively). None of the four hormones were found to change significantly throughout the year (n = 5, CC: Friedman chi-squared = 2.8286, p = 0.7264; E2: ANOVA all factors p > 0.05; P4: ANOVA all factors p > 0.05; T: ANOVA all factors p > 0.05).

## Discussion

In this study, we utilized a captive, breeding colony of oviparous snakes (Louisiana pinesnake, *Pituophis ruthveni*, LPS) to measure hormones across the active season from both fecal and plasma samples. We analyzed both sample types for corticosterone, estradiol, progesterone, and testosterone (CC, E2, P4, and T respectively) levels and have attempted to correlate the changes in hormone levels with time of year and specific life history stages (e.g. post-brumation, breeding, post-breeding)

Corticosterone is the main glucocorticoid in snakes [29] and has been shown to have a positive correlation with T levels in some viviparous snake species, increasing during gametogenesis and declining over the breeding season (*Thamnophis sirtalis concinnus*, [30]). In male LPS, circulating CC was elevated during Breeding, but this elevation was only significant compared to Post-Brumation levels (Fig 1A; 7.72 ± 0.75 and 3.80 ± 0.44 ng/mL respectively, p = 0.0058) and otherwise CC levels were higher than Post-Brumation, but highly variable and showed no significant decline after breeding. Unlike CC, male plasma T was elevated briefly during Breeding (Fig 1B; 56.6 ± 11.51 ng/mL) before falling to baseline levels (1.5 to 3.1ng/mL) throughout the rest of the active season. The patterns here support the role of T in breeding, but, interestingly, not during the likely period of gametogenesis. Based on semen collections performed throughout the year, the highest quality and quantity of sperm is available during the Post-Brumation, Breeding, and late Fall/Pre-Brumation periods (pers.obs) indicating that this species likely undergoes post-nuptial spermatogenesis, without significant increases in circulating T, and subsequently stores that sperm overwinter for use the following spring. This hormone pattern, or lack thereof, is similar to what has been seen in male Western diamond-backed rattlesnakes, where CC levels did not vary with season and were not correlated with T levels and T levels were increased only during breeding season (*Crotalus atrox*, [31]).

While we anticipated finding seasonal patterns in female LPS hormones, only a significant decrease in circulating P4 levels immediately post-lay was found. Female LPS showed no seasonal pattern in CC, E2, or P4 (Fig 1E-H; Fig 2E-H). In other oviparous reptiles, there is a synchroneity in hormones allowing the inhibitory effects of P4 on hepatic and reproductive tract function to be offset by the stimulatory effects of E2 ensuring the appropriate secretion of vitellogenin and reproductive tract development [13]. This synchrony includes a pre-ovulatory peak in E2, T, and P4 (*Chyrsemys picta*, [32]); unlike what has been reported for oviparous lizards which have a post-ovulatory peak in E2, T, and P4 [33,34]. In a viviparous snake, P4 levels increased prior to ovulation, and remained elevated throughout pregnancy only decreasing close to parturition (*V. aspis*, [35]). While we did see a steep decline in plasma P4 levels immediate post-lay (Fig 1H), we did not find a corresponding peak in CC, E2, or P4 that could potentially coincide with ovulation. Since we cannot conclusively identify the timing of ovulation, we have previously relied on ultrasonography to determine the development stage of follicles in this species [36] and found no hormonal changes that correspond to the development of follicles from the pre-vitellogenic stage (Day 0–89, Post-Brumation) to vitellogenesis (Day 103–139, Breeding).

The collection of blood samples is the most common way to monitor hormones and other physiological parameters; however, blood sampling requires the handling of animals which induces stress and is limited by volume, frequency of collections, and timing of collections to ensure the health and safety of the animals (e.g., not restraining heavily gravid females nor separating actively breeding pairs). These limitations resulted in lower sample sizes and may have contributed to a lack of statistical differences reported here.” Monitoring steroid hormones in fecal samples has become accepted as a non-invasive way to monitor physiological changes in animals (reviewed in [37]) and fecal metabolite levels have been shown to track circulating hormone patterns in a number of species (e.g. goats [38]; hare [39]; fowl: [40]). Although the measurement of glucocorticoid and reproductive hormone metabolites in feces has become an accepted method for the noninvasive evaluation of adrenocortical and gonadal activity for many species, the use of fecal hormone monitoring has been underutilized in reptiles to date. We found that fecal samples provided similar patterns of hormones to plasma samples. This finding provides positive evidence for the use of non-invasive sampling of snakes to measure hormones. This might be a useful strategy in species that do not do well with frequent handling, are dangerous to handle (e.g. highly venomous), or are difficult to bleed. However, collection of fecal samples was not reliable throughout the year and might be limited to use in species that feed and defecate regularly, especially during reproductively important times of the year when hormone levels are of greatest interest. Fecal samples were collected from cages opportunistically which resulted in very few individuals that had samples collected during each of the time bins analyzed (for females 5 of 23 individuals in our population had qualifying samples and for males 4 of 20 individuals could be included). The snakes in our collection defecate ~weekly, depending on how often they eat which varies across the active season (e.g. males frequently refuse to eat during the breeding season, females do not eat for weeks when they are heavily gravid, and individuals of both sexes can exhibit anorexia prior to the start of brumation; pers. obs). This low success rate, especially during certain times of the year, resulted in low statistical power that likely contributed to the lack of significant differences reported here.

This study is, to our knowledge, the first and most comprehensive report on hormone levels across the active season of any oviparous snake. While members of the reptile taxon are universally understudied compared to other taxa, oviparous snakes have been particularly underrepresented and the data presented here are a beginning towards understanding hormone cycles in oviparous snakes. Our study is also one of few to compare different sample types (feces vs. plasma) and to demonstrate the advantages and disadvantages of collection methods in a reptile.

## Supporting information

S1 Female LPS Fecal Data. Contains the raw data derived from fecal samples collected from female Louisiana pinesnakes.
(CSV)

S2 Female LPS Plasma Data. Contains the raw data derived from plasma samples collected from female Louisiana pinesnakes.
(CSV)

S3 Male LPS Fecal Data. Contains the raw data derived from fecal samples collected from male Louisiana pinesnakes.
(CSV)

S4 Male LPS Plasma Data. Contains the raw data derived from plasma samples collected from male Louisiana pinesnakes.
(CSV)
